# Anti-Mycobacterial Activity of Flavonoid and Pyrimidine Compounds

**DOI:** 10.3390/molecules27196714

**Published:** 2022-10-09

**Authors:** Saurabh Garg, Rakesh Kumar, Dennis Kunimoto, Gina R. Rayat

**Affiliations:** 1 Department of Surgery, Ray Rajotte Surgical-Medical Research Institute, Alberta Diabetes, and Transplant Institutes, Faculty of Medicine and Dentistry, College of Health Sciences, University of Alberta, Edmonton, AB T6G 0X0, Canada; 2 ImMed Biotechnologies, Edmonton, AB T6R 2E8, Canada; 3 Department of Medicine, Faculty of Medicine and Dentistry, College of Health Sciences, University of Alberta, Edmonton, AB T6G 2R7, Canada

**Keywords:** tuberculosis, pyrimidines, flavonoid, mycobacteria

## Abstract

We evaluated the anti-mycobacterial effect of a flavonoid 5,7-dihydroxy-2-(4-hydroxyphenyl) 4*H*-chromen-4-one (**1**) and two pyrimidines, 4-hydroxy-2-dimethylamino-5-nitroso-6-aminopyrimidine (**2**) and 2-chloro-5-*n*-nonylpyrimidine (**3**) in vitro against *Mycobacterium tuberculosis* (*M. tuberculosis*, H37Ra) and *Mycobacterium avium (M. avium)*, using a Microplate Alamar Blue Assay (MABA). The effects of the compounds **1**–**3** in combination with first- and second-line anti-TB drugs isoniazid, rifampicin, cycloserine, and clarithromycin on the growth of *M. tuberculosis* and *M. avium* were also evaluated in in vitro assays. As a single agent, compounds **1** and **2** exhibited modest activity while compound **3** was the most effective against *M. tuberculosis* and *M. avium*. When compounds **1**–**3** were evaluated at lower than 50% of their inhibitory concentrations in a two-drug combination with isoniazid or rifampicin, they showed additive to synergistic interactions. This inhibitory effect was improved when each of the three compounds was tested together in a three-drug combination with two of the first-line anti-TB drugs. Compounds **1**–**3** also demonstrated strong synergistic interaction in combination with cycloserine and clarithromycin in inhibiting the growth of *M. tuberculosis* and *M. avium*, respectively. This study demonstrated that compounds **1**–**3** have potential to be developed as effective anti-TB agents with combined use.

## 1. Introduction

Tuberculosis (TB) is an infectious disease transmitted through the air by sneezing and coughing. TB is caused by several species of the bacterial genus *Mycobacterium* [1,2,3] and has become one of the leading causes of death worldwide [1]. In the year 2020, the World Health Organization reported 10 million people developed TB and 1.4 million died from this disease. In addition, 0.21 million died from TB–HIV co-infection [2,3]. Nearly one-quarter of the global population, an estimated 1.7 billion people, are infected with *Mycobacterium tuberculosis* (*M. tuberculosis*, *Mtb*) and hence are at risk of developing TB during their lifetime [2,3,4]. People with compromised immune systems due to conditions such as diabetes, HIV infection, or malnutrition are more prone to develop TB [2,3,4,5,6]. *M. tuberculosis* is highly contagious and poses a fatal threat to immunocompromised HIV patients [7,8].

TB is a complex disease, localized in the lungs or disseminated throughout the body, and is caused by any one of the groups of mycobacteria known as the *Mycobacterium tuberculosis* complex (MTBC) and the *Mycobacterium avium (M. avium)* complex (MAC) [9,10]. The MTBC group includes *M. tuberculosis*, *M. bovis*, *M. microti*, *M. africanum*, *M. caprae*, and *M. pinnipedii*. Members of MTBC are extremely pathogenic to humans and some animals and can cause serious lung disease. The *Mycobacterium avium (M. avium)* complex (MAC) comprises *M. avium* and *M. intracellulare* [11]. The bacteria of the MAC group are environmental microbes that are usually not pathogenic in humans but cause serious lung or disseminated disease, especially in patients with compromised immune systems, such as those with HIV/AIDS, cancer, diabetes, and other underlying diseases [12].

Due to the lack of an effective vaccine, drug therapy remains the major tool in controlling the disease. The first-line anti-TB drugs that form the core of treatment regimens include isoniazid, rifampicin, pyrazinamide, and ethambutol. Isoniazid (INH) is a prodrug that is activated by mycobacterial catalase–peroxidase (KatG). Upon activation, INH inhibits the enoyl-acyl carrier protein reductase known as InhA, resulting in the inhibition of mycolic acid synthesis, which hinders the formation of the mycobacterial cell wall, and in turn, inhibits mycobacterial growth [13,14]. Rifampicin (RIF) inhibits bacterial DNA transcription to RNA by binding to the β-subunit of DNA-dependent RNA polymerase in bacterial cells, thereby inhibiting protein synthesis and possessing bactericidal activity [9,10,13,14]. Pyrazinamide (PZ) is a prodrug that is converted into the active drug pyrazinoic acid by *M. tuberculosis* pyrazinamidase. The pyrazinoic acid then inhibits the enzyme fatty acid synthase I, resulting in the inhibition of cell wall component synthesis, mycolic acid [15,16]. Ethambutol (EMB) interferes with the synthesis of the mycobacterial cell wall component arabinogalactan by inhibiting the enzyme arabinosyl transferase [13,14,15,16].

The emergence of drug resistance in *M. tuberculosis* strains raises severe concerns for available drug therapy. Multidrug-resistant (MDR) TB is characterized by the resistance to first-line TB drugs, rifampicin, and isoniazid. Extensively drug-resistant (XDR) TB refers to a resistance to rifampicin and isoniazid, as well as any fluoroquinolone and at least one of the three injectable second-line drugs, kanamycin, amikacin, and capreomycin [17,18]. The standard short-course chemotherapy for TB consists of a combination of TB drugs with six months duration. This regimen produces an 85–90% success rate in 100% compliant individuals. However, patients with MDR-TB are treated with a regimen of a combination of four–five drugs for greater than 15 months after a negative culture is obtained. This duration, accompanied by frequent serious side effects, leads to unsatisfactory patient compliance, and cessation of treatment [19,20]. Many second-line drugs (e.g., ethionamide, PAS, cycloserine, and kanamycin) are less preferred because of their lower efficacy and more serious side effects [19,20,21,22].

Flavonoid and pyrimidine analogs comprise a major class of rationally designed agents for the treatment of several viral and cancer diseases [23,24,25,26,27,28]. These classes of compounds have also been explored for their antibacterial activity [29,30,31,32,33]. Previous research has shown the antibacterial activity of flavonoids via different mechanisms [34]. Ohemeng et al. reported that flavonoids specifically inhibit the DNA gyrase of *E. coli* and *S. aureus* [35]. Meanwhile, studies by Ikigai et al. and Plaper et al. have suggested that the antibacterial activity of flavonoids is due to cytoplasmic membrane damage [36,37]. Avila et al. reported the inhibitory effect of flavonoids on energy metabolism and proposed that the lipophilicity of ring A flavonoids is crucial for their antibacterial activity [38,39]. Mori et al. have also shown the antibacterial effect of flavonoids through the inhibition of nucleic acid synthesis [40,41], while Wu et al. have reported the antibacterial activity of quercetin and apigenin via the inhibition of cell wall synthesis [42]. It has also been reported that the hydroxyl group in the B-ring of flavonoids stabilized the triple-stranded DNA/RNA and duplex RNA/DNA structures [43,44,45].

**Figure 1:**
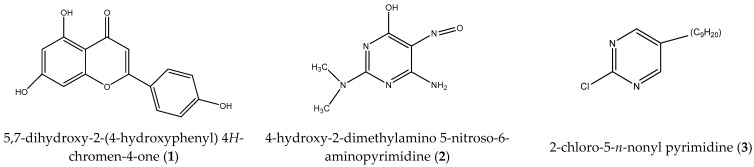
Chemical structure of compounds **1**, **2**, and **3**.

The complete genome sequence analysis of *M. tuberculosis* has advanced the development of novel targets for new anti-TB drugs [33,46,47,48]. Several enzymes involved in pyrimidine and purine biosynthesis and metabolism, as well as DNA and RNA synthesis differ significantly between *M. tuberculosis* and human [49,50]. This suggests that modified pyrimidine analogs may have the potential to interfere with these targets by disrupting nucleic acid biosynthetic and metabolic pathways in mycobacteria. Consequently, pyrimidine analogs have also demonstrated significant anti-TB activity [51,52]. Previous studies have shown that 5-alkynated groups in pyrimidine analogs are more hydrophobic than methyl group present in thymidine, facilitating their incorporation into DNA and RNA structures as well as their intake in bacterial cells [53,54]. Thus, the C-5 position of pyrimidine nucleosides is an attractive target for modification [55]. Such modification has been incorporated in two pyrimidines used in this study. As such, we have selected a flavonoid compound 5,7-dihydroxy-2-(4-hydroxyphenyl 4*H*-chromen-4-one) and two pyrimidine compounds 4-hydroxy-2-dimethylamino-5-nitroso-6-aminopyrimidine and 2-chloro-5-*n*-nonylpyrimidine, which we will refer to as compounds **1**, **2**, and **3**, respectively (Figure 1), as anti-TB agents. Based on previous findings, we speculate that these compounds will inhibit *M. tuberculosis* replication by selectively incorporating into the mycobacterial DNA/RNA chain and/or inhibiting bacterial cell wall synthesis. Compounds **1**, **2**, and **3** have not been explored previously as single anti-TB agents or in combination with the current conventional anti-TB drugs. Therefore, in this study, we evaluated the ability of the three compounds alone and in combination with current anti-TB drugs isoniazid, rifampicin, and cycloserine to inhibit the growth of *M. tuberculosis* (H37Ra). In addition, the efficacy of the three compounds alone and in combination with clarithromycin to inhibit the growth of *M.* *avium* was assessed.

## 2. Results and Discussion

The anti-mycobacterial activity of compounds **1**–**3** was evaluated in vitro against *M. tuberculosis* (H37Ra) using a Microplate Alamar Blue Assay (MABA) at concentrations of 1.56 to 200 µg/mL (Table 1). Anti-TB drugs, isoniazid, rifampicin, and cycloserine, were used as standard anti-TB drugs. Compound **1**, a flavonoid, displayed 85% inhibition at 200 µg/mL, 73% at 100 µg/mL, and 53% at 50 µg/mL. Compound **2**, a pyrimidine analog that contains 5-nitroso at the C-5 position, provided 99% inhibition at 200 µg/mL, 96% at 100 µg/mL, and 52% at 50 µg/mL. Compound **3**, a pyrimidine analog that contains a longer carbon chain at the C-5 position, was found to be most effective at inhibiting the growth of *M. tuberculosis*. This compound demonstrated a dose-dependent inhibition of *M. tuberculosis* growth by 99%, 97%, and 92% at concentrations of 200, 100, and 50 µg/mL, respectively.

Previous studies have shown that when used in combination, drugs with low efficacy have therapeutic effects, even against resistant strains. Combined drug treatment is a standard treatment strategy for numerous chronic infections, including *M. tuberculosis*. Combination therapy not only lowers the therapeutic dose of a single drug but may also prevent the development of resistance against each individual drug. To elucidate any potential and likely synergistic interactions of our three compounds with current anti-mycobacterial drugs, we evaluated the anti-mycobacterial activity of compounds **1**, **2**, and **3** in combination with the first-line anti-TB drugs, isoniazid and rifampicin, as well as a second-line anti-TB drug, cycloserine, on *M. tuberculosis* (H37Ra) in vitro. The MABA assay was used to evaluate the effect of compounds **1**, **2**, and **3** at 1.56 µg/mL to 200 µg/mL concentrations alone or in combination with isoniazid at 0.2 µg/mL (<MIC_50_), rifampicin at 0.26 ng/mL (MIC_50_), and cycloserine at 5.5 µg/mL (<MIC_50_).

**Figure 2:**
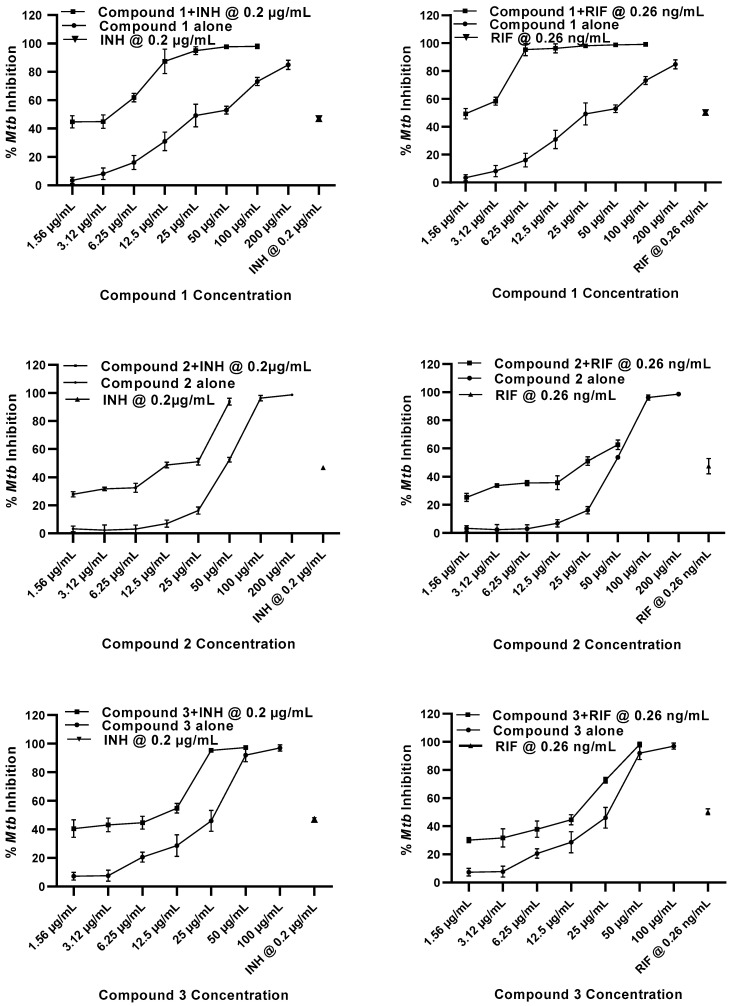
In vitro combination effect of compounds **1**, **2**, and **3** with isoniazid (INH) and rifampicin (RIF) against *M. tuberculosis* (H37Ra). Anti-mycobacterial activity was determined at 200, 100, 50, 25, 12.5, 6.25, 3.12, and 1.56 µg/mL concentrations. Positive control drugs isoniazid at 0.2 µg/mL and rifampicin at 0.26 ng/mL were used. The experiments were performed on three separate days, in triplicate each day, and the mean percent inhibition was calculated.

Isoniazid exhibits its anti-mycobacterial activity by inhibiting mycolic acid synthesis, thereby inhibiting cell wall synthesis [56,57]. Isoniazid alone showed a 47% inhibition of *M. tuberculosis* growth at 0.2 µg/mL, whereas compounds **1** and **3** exhibited 49% and 46% inhibition at 25 µg/mL, respectively (Figure 2, Table 2). When compounds **1** and **3** were combined at 25 µg/mL with isoniazid at 0.2 µg/mL, an additive effect was observed, resulting in 98% and 95% growth inhibition of *M. tuberculosis*, respectively (Figure 2) with a combination index (CI) of 1.0. (Table 2). At 12.5 µg/mL, compound **1** is slightly in synergy with isoniazid (CI = 0.9). Compound **2** alone at 50 µg/mL had 52% inhibition but showed an additive effect with a 94% inhibition of *M. tuberculosis* growth when combined with isoniazid at 0.2 µg/mL (CI = 1) (Figure 2, Table 2). These results suggest that compounds **1** and **3** may be acting on different targets than isoniazid. As such, when combined at low doses, the efficacy of compounds **1** and **3** as well as isoniazid in inhibiting the growth of *M. tuberculosis* (H37Ra) is elevated compared to their individual anti-mycobacterial effects.

Rifampicin inhibits DNA-dependent RNA polymerase by binding to the β-subunit, thereby inhibiting bacterial DNA transcription to RNA and subsequent translation to proteins [57,58]. Rifampicin alone showed a 50% anti-mycobacterial activity at 0.26 ng/mL while compound **1** alone displayed only a 16% inhibition at 6.25 µg/mL and a 49% inhibition at 25 µg/mL, respectively (Figure 2, Table 2). Interestingly, compound **1** showed a synergistic effect at both concentrations when combined with rifampicin, resulting in a 95% to 98% growth inhibition of *M. tuberculosis* with CI values of 0.6 and 0.9 (Table 2). The activity of compounds **2** and **3** at 50 µg/mL and 25 µg/mL, respectively, was slightly improved in combination with rifampicin, with 63% and 73% inhibition against *M. tuberculosis*, respectively. These results suggest that only the flavonoid compound **1**, but not pyrimidine analogs **2** and **3**, has a strong anti-mycobacterial effect on *M. tuberculosis* in combination with rifampicin. The exact mechanism of this synergistic effect is currently not known; however, it is possible that while rifampicin is inhibiting RNA synthesis, compound **1** is inhibiting bacterial cell wall synthesis.

**Figure 3:**
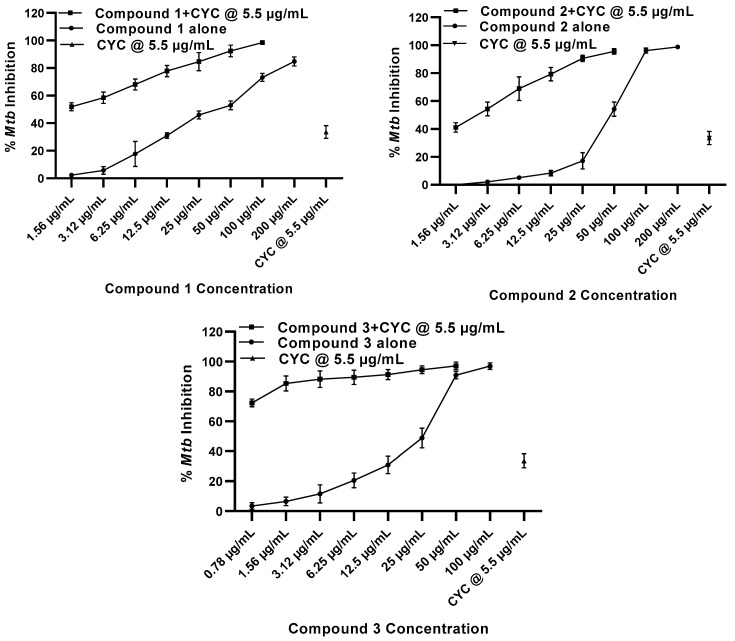
In vitro combination effect of compounds **1**, **2**, and **3** with cycloserine (CYC) against *M. tuberculosis* (H37Ra). Anti-mycobacterial activity was determined at 200, 100, 50, 25, 12.5, 6.25, 3.12, and 1.56 µg/mL concentrations. Positive control drug cycloserine (CYC) at 5.5 µg/mL was used. The experiments were performed on three separate days in triplicate each day and the mean percent inhibition was calculated (mean ± SD).

The second-line anti-TB drug, cycloserine, is mainly used to treat MDR and XDR TB. Its mechanism of action is not known, but it has been suggested that cycloserine inhibits peptidoglycan synthesis, which is crucial for the mycobacterial cell wall development, thus inhibiting mycobacterial growth [13,59,60]. Cycloserine alone had a 34% inhibition of *M. tuberculosis* at 5.5 µg/mL (Figure 3, Table 3). However, a synergistic effect was observed when cycloserine was combined with compounds **1**–**3**. The combination of cycloserine at 5.5 µg/mL and compound **1** at 25 µg/mL and 50 µg/mL resulted in 85% and 92% inhibition, respectively, in comparison to 49–53% inhibition by compound **1** alone at these concentrations. When cycloserine was combined with compound **2** at the same concentrations, 91% and 96% inhibition was observed, respectively, whereas compound **2** alone had 54% (at 50 µg/mL) and 17% (at 25 µg/mL) inhibition (Figure 3, Table 3). The combination of cycloserine at 5.5 µg/mL with compound **3** at 1.56 µg/mL to 25 µg/mL concentrations exhibited 85% to 95% inhibition in contrast to 7–49% inhibition by compound **3** alone (Figure 3, Table 3). It is worth noting that even at lower concentrations (≤1.56 µg/mL) of the three compounds, synergism with cycloserine with a CI of ≤0.8 was still observed (Table 3). The mechanism behind the synergistic effect of these anti-TB agents is currently not known. However, it is possible that cycloserine may weaken the lipophilic cell wall of *M. tuberculosis*, increasing the uptake of the three compounds and thus improving their anti-mycobacterial activity at low doses.

**Figure 4:**
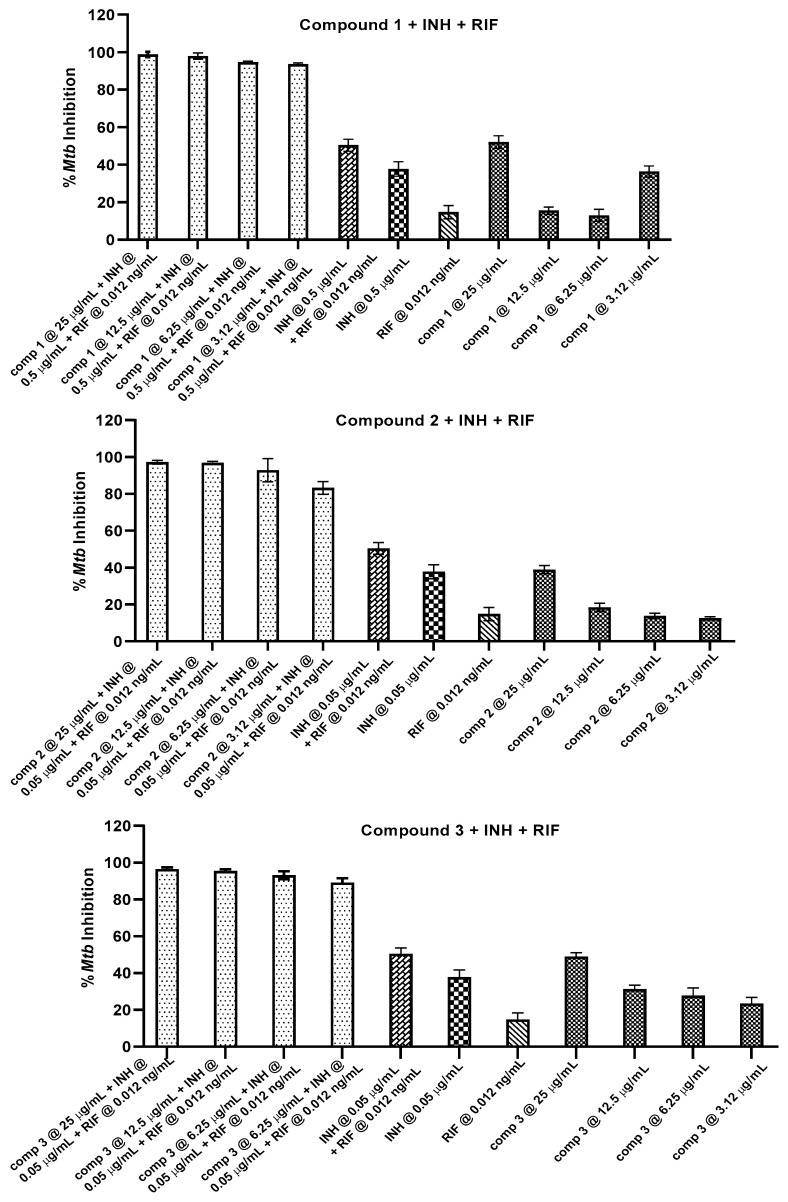
In vitro combination effect of compounds **1**, **2**, and **3** with isoniazid (INH) and rifampicin (RIF) against *Mtb* (H37Ra). Anti-mycobacterial activity against *Mtb* (H37Ra) of compounds **1**, **2**, and **3** was determined at concentrations of 25, 12.5, 6.25, and 3.12 µg/mL in combination with isoniazid (0.05 µg/mL) and rifampicin (0.012 ng/mL). The experiments were performed on three separate days, in triplicate on each day and the mean percent inhibition was calculated.

Encouraged by the outcomes from the two-drug combinations, we then evaluated the efficacy of compounds **1**, **2**, and **3** in a three-drug combination in inhibiting mycobacterial growth (Figure 4). To determine any possible interaction of compounds **1**, **2**, and **3** with the current anti-mycobacterial drugs, we tested compounds **1**–**3** at less than 50% inhibitory concentration ranges of 3.12 µg/mL to 25 µg/mL, with isoniazid at 0.05 µg/mL and rifampicin at 0.012 ng/mL. All three compounds showed a ≥83% inhibition at 3.12 µg/mL to 25 µg/mL, and were synergistic with the two standard first-line anti-TB drugs in inhibiting the growth of *M. tuberculosis*. The CI values obtained for compounds **1**, **2**, and **3** in the three-drug combination range from 0.6 to 1.0 (Table 4). In these three-drug combination studies, we noted that the activity obtained upon combining the three compounds with isoniazid and rifampicin was significantly higher than the two drugs isoniazid and rifampicin combined together, which obtained 50% inhibition. These results indicate the vital contribution of compounds **1**, **2**, and **3** in providing enhanced anti-mycobacterial activity. The precise mechanism of synergism is unclear but may be attributed to simultaneously targeting three distinct critical components for mycobacterial survival, i.e., the disruption of cell wall synthesis, the inhibition of RNA synthesis, and nucleic acid synthesis.

**Figure 5:**
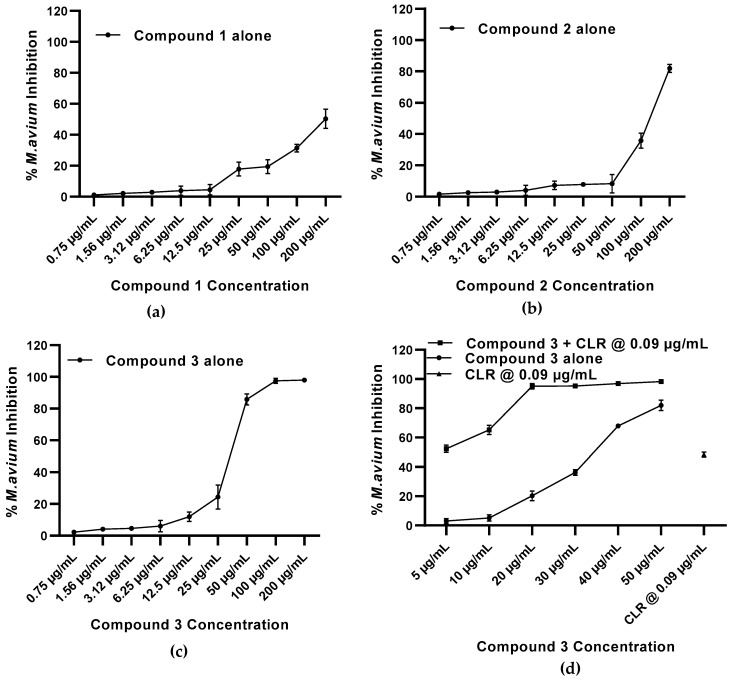
In vitro effect of compounds **1**, **2**, and **3** against *M. avium*. Drug dose response of compounds **1**, **2**, and **3** were determined at concentrations 200, 100, 50, 25, 12.5, 6.25, 3.12, 1.56, and 0.75 µg/mL against *M. avium* (**a**–**c**). For the two-drug combination, compound **3** alone and in combination with clarithromycin (CLR) was determined at 50, 40, 30, 20, 10, and 5 µg/mL concentrations against *M. avium* (**d**). The positive control drug clarithromycin at 0.09 µg/mL was used. The experiments were performed on three separate days in triplicate each day and the mean percent inhibition was calculated.

We wondered whether our three compounds would also be equally efficacious in inhibiting the growth of the MAC group strain of mycobacteria. Thus, we evaluated the effect of compounds **1**, **2**, and **3** against *M. avium*. When compounds **1**, **2**, and **3** were tested against *M. avium*, compounds **1** and **2** displayed appreciable activity (50% and 82% growth inhibition, respectively) at the highest drug concentration tested (200 µg/mL). In contrast, compound **3** showed 98%, 97%, and 86% inhibition of *M. avium* growth at 200, 100, and 50 µg/mL concentrations, respectively (Figure 5, Table 5). Clarithromycin (CLR), in these assays, showed a 99% inhibition of *M. avium* growth at 1 µg/mL and was used as a standard drug for *M. avium* [61]. Clarithromycin has a minimum activity against *M. tuberculosis* but has been shown to have a synergistic effect against multi-drug resistant strains of *M. avium* when used in combination with other anti-TB drugs [62]. Therefore, we also evaluated the effect of the combination of the most active compound **3** with clarithromycin at doses that exhibit ≤50% growth inhibition of *M. avium*. At 5 µg/mL to 30 µg/mL, compound **3** alone demonstrated the lowest anti-*M. avium* activity with 3% to 36% inhibition, respectively (Table 6). However, when compound **3** was combined at these concentrations with clarithromycin at 0.09 µg/mL (48% growth inhibition), a synergistic effect was observed (52% to 95% inhibition of *M. avium* growth) with a CI of 0.7–0.9 (Table 6). The synergistic interaction noted could possibly be due to the simultaneous inhibition of the two distinct key components, i.e., nucleic acid synthesis by compound **3** and protein synthesis by clarithromycin, which together contribute to the substantial decline in mycobacterial growth.

**Figure 6:**
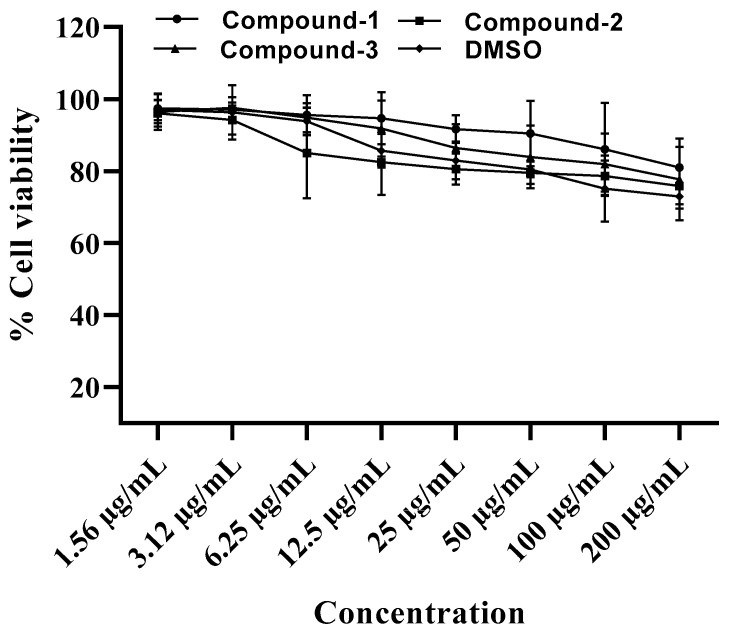
In vitro toxicity of compounds **1**, **2**, and **3** on Vero cells. The CCK8 assay was performed to evaluate the toxicity of the test compounds at concentrations of 200, 100, 50, 25, 12.5, 3.12, and 1.56 µg/mL toward Vero cells. DMSO was used as a control. The experiment was repeated three times and the mean percent cell viability is provided.

The toxicity of compounds **1**, **2**, and **3** was performed using a CCK8 assay in Vero cells. As shown in Figure 6, the viability of the Vero cells was not significantly affected by any of the three compounds, even at the highest concentration (CC_50_ > 200 μg/mL) tested.

Taken together, this study demonstrated that newly identified flavonoid 5,7-dihydroxy-2-(4-hydroxyphenyl) 4*H*-chromen-4-one (compound **1**) and two pyrimidines, 4-hydroxy-2-dimethylamino-5-nitroso-6-aminopyrimidine (compound **2**) and 2-chloro-5-*n*-nonyl pyrimidine (compound **3**), possess significant anti-mycobacterial activities, albeit at high doses; however, they exhibit additive and/or synergistic interactions when combined with other anti-TB agents that target different pathways leading to the potent inhibition of both *M. tuberculosis* and *M. avium,* even at their low doses. Thus, compounds **1**, **2**, and **3** are capable of reducing the doses of established anti-TB drugs with synergistic contributions and may be effective at preventing the emergence of resistance against the current anti-TB drugs.

## 3. Materials and Methods

### 3.1. Synthesis of Compound ***1***

The method as described by Gothelf et al. [63] was exactly followed for the synthesis of compound **1**. Briefly, 3-(4-hydroxyphenyl)-5-tributylstannylisoxazole was first synthesized by stirring a reaction mixture comprising potassium hydrogen carbonate, water, tributylstannyl acetylene, *N*-chlorosuccinimide, and 4-hydroxybenzaldehyde oxime in ethyl acetate for ~20 h at room temperature. The desired product was purified by silica gel column chromatography. In the next step, 3-(4-hydroxyphenyl)-5-(2,4,6-trihydroxyphenyl)isoxazole was synthesized by heating 3-(4-hydroxyphenyl)-5-tributylstannylisoxazole and palladium (II) chloride in anhydrous dioxane to 105 °C under nitrogen followed by the addition of iodophloroglucinol in anhydrous dioxane. The reaction mixture, after refluxing for 3 h, was filtered, evaporated, and purified on a silica gel column. In the final step, the target compound **1** was synthesized by catalytic reduction of 3-(4-hydroxyphenyl)-5-(2,4,6-trihydroxyphenyl)isoxazole with Raney-Ni in aqueous methanol and boric acid, followed by refluxing of the reduced product in presence of AcOH and concentrated hydrochloric acid for 1 h, and recrystallization of the obtained residue from ethanol. Compounds **2** (Product number-H0562) and **3** (CAS number-219581-06-03) were purchased from TCI America Inc., Portland, OR, USA, and Toronto Research Chemicals Inc., North York, ON, Canada, respectively. 

### 3.2. In Vitro Anti-Mycobacterial Activity Assay

*M. tuberculosis* (H37Ra) (ATCC 25291) and *M. avium* (ATCC 25291) were obtained from the American Type Culture Collection (ATCC, Rockville, MD, USA). Both strains were cultured in Middlebrook 7H9 Broth medium supplemented with glycerol, Tween 80, and Middlebrook enrichment, which contain bovine albumin, dextrose, and catalase (Becton Dickinson Co., MD, USA). The anti-mycobacterial activity was determined using the MABA assay. The cell viability reagent alamarBlue^®^ was purchased from Bio-Rad Laboratories, Inc. (Mississauga, ON, Canada). Test compounds were dissolved in dimethyl sulfoxide (DMSO) at 10 mg/mL and subsequent dilutions were made in 7H9GC medium in 96-well plates. For these experiments, each compound was tested at 200, 100, 50, 25, 12.5, 6.25, 3.12, and 1.56 µg/mL in triplicate. Frozen mycobacterial inoculates were diluted in a 7H9GC medium and added to each well at a final concentration of 2.5 × 10^5^ CFU/mL. Six wells with medium alone (M) and six wells with bacteria alone (B) were used as controls. Plates were incubated for six days and then 20 µL of 10× alamarBlue^®^ and 12.5 µL of 20% Tween 80 were added to one M and one B well. Wells were observed for an additional 24–48 h for visual color change from blue to pink and absorbance values were determined at excitation 530/525 nm and emission 590/535 nm using a spectrophotometer (Envision 2104 Multilabel Reader, PerkinElmer, Waltham, MA, USA). If the B well became pink by 24 h (indicating growth), reagent was added to the entire plate. If the B well remained blue, additional M and B wells were tested daily until bacterial growth could be visualized by color change. After the addition of the reagent to the plate, cultures were incubated for 24 h, plates were observed visually for color change, absorbance values were determined as previously described. Minimum inhibitory concentration (MIC) was determined visually as the lowest concentration of a compound that prevented a color change from blue to pink. Percent inhibition was calculated as 100 − (test well − medium well)/(bacteria well − medium well) × 100. Similar methodology was used for *M. avium* strain. Isoniazid, rifampicin, cycloserine, and clarithromycin were used as positive controls. As negative controls, DMSO was added to the B well at concentrations similar to those of compounds **1**, **2**, and **3**; M wells also served as negative controls. In most of the experiments, the M wells gave absorbance values of 6000 to 8000, and the B wells had absorbance values ranging from 90,000 to 130,000.

### 3.3. In Vitro Anti-Mycobacterial Activity of Test Compounds in Combination with Standard Drug(s)

For drug combination studies, MABA was used utilizing similar methodology as described above. For the two drug combination studies, isoniazid (Sigma-Aldrich, St. Luis, MO, USA) at 0.2 µg/mL, rifampicin (Sigma-Aldrich) at 0.026 ng/mL, cycloserine (Sigma-Aldrich) at 5.5 µg/mL, and clarithromycin (Sigma-Aldrich) at 0.09 µg/mL concentrations were used. For the three-drug combination studies, isoniazid at 0.05 µg/mL and rifampicin at 0.012 ng/mL concentrations, respectively, were used. The combination effect of compounds was determined by calculating the combination index (CI). CI was calculated as the sum of the individual drug effect divided by the combination effect of the drugs. If CI values were <1.0, this indicated a synergistic effect (i.e., the combined effect was greater than the sum of their individual effects). If CI = 1.0, this indicated an additive effect (i.e., the combined effect was equal to the sum of their individual effects) and if CI >1.0, this indicated an antagonistic effect (i.e., the combined effect was less than the sum of their individual effects) [64,65,66].

### 3.4. In Vitro Cytotoxicity Assay

Cell viability was measured using the cell counting Kit-8 (CCK-8, Abcam Inc., Cambridge, MA, USA). A 96-well plate was seeded with Vero cells (ATCC, CCL-81™) cultured in Dulbecco’s Modified Eagle Medium (DMEM, Gibco Thermo Fisher Scientific, Grand Island, NY, USA) supplemented with 10% heat-inactivated fetal bovine serum (Gibco Thermo Fisher Scientific, Grand Island, NY, USA) at a density of 2 × 10^5^ cells per well. Compounds were dissolved in DMSO at 10 mg/mL and subsequent dilutions were made in DMEM medium in 96-well plates. Cells were allowed to attach for 24 h, and the DMEM medium was replaced with DMEM medium containing compounds at concentrations of 200, 100, 50, and 10 µg/mL. DMSO was also included as solvent control. Plates were incubated for 3 days at 37 °C. The color reaction involved adding 10 µL of CCK-8 dye reagents per well and incubating for 4 h at 37 °C until the color of the reagent changed to orange. Absorbance were measured using an ELISA plate reader (Envision 2104 Multilabel Reader, PerkinElmer, Akron, OH, USA) at the wavelength of 450–500 nm. Percent viability was calculated as (absorbance of test well) − (absorbance of medium well without cells)/(absorbance of control solvent well) − (absorbance of medium well without cells) × 100.

### 3.5. Data Analyses

Data analyses were performed using GraphPad Prism ver. 8.0.0 for windows (GraphPad Software, San Diego, CA, USA). Data were represented as mean ± standard deviation (SD).

## Figures and Tables

**Table 1:** In vitro anti-mycobacterial activity of compounds **1**, **2**, and **3** against *M. tuberculosis* (H37Ra).

Compounds	Anti-Mycobacterial Activity ^a,b^ % Inhibition (Concentration, µg/mL)
	*M. tuberculosis (H37Ra)*
**1**	85 ± 3.30 (200), 73 ± 2.90 (100), 53 ± 2.98 (50), 49 ± 4.18 (25)
**2**	99 ± 0.78 (200), 96 ± 1.90 (100), 52 ± 1.65 (50), 16 ± 2.59 (25)
**3**	99 ± 1.23 (200), 97 ± 2.18 (100), 92 ± 4.58 (50), 46 ± 7.32 (25)
Isoniazid^c^	99 ± 2.97 (1)
Rifampicin^c^	99 ± 1.06 (0.5)

^a^ Anti-mycobacterial activity was determined at concentrations of 200, 100, 50, 25, 12.5, 6.25, 3.12, and 1.56 µg/mL. ^b^ Average from three independent repeated experiments ± SD. ^c^ Positive control TB drugs.

**Table 2:** In vitro anti-mycobacterial activity and combination index (CI) of compounds **1**, **2**, and **3** in a two-drug combination with isoniazid and rifampicin.

		Anti-Mycobacterial Activity *Mtb* (H37Ra)						
Compound	Concen-tration (µg/mL)	Compound Alone	Combination with 0.2 µg/mL INH			Combination with 0.26 ng/mL RIF		
		% Inhibition	% Inhibition	CI ^a^	Outcome	% Inhibition	CI ^a^	Outcome
**1**	50	53 ± 1.96	98 ± 3.23	1.0	Additive	98 ± 2.41	1.0	Additive
	25	49 ± 7.78	98 ± 2.98	1.0	Additive	98 ± 3.21	0.8	Synergistic
	12.5	31 ± 6.61	96 ± 8.61	0.9	Synergistic	96 ± 3.22	0.6	Synergistic
	6.25	16 ± 4.96	95 ± 3.20	1.0	Additive	95 ± 4.43	0.9	Synergistic
**2**	50	53 ± 1.65	94 ± 2.40	1.0	Additive	63 ± 3.39	-	-
**3**	25	46 ± 7.32	95 ± 3.00	1.0	Additive	73 ± 2.12	-	-
INH	0.2	47 ± 2.78	-	-	-	-	-	-
RIF	0.00026	50 ± 2.14	-	-	-	-		-

^a^ CI < 1 (Synergistic), 1 (Additive). The positive control drugs INH (isoniazid) and RIF (rifampicin) were used at 0.2 µg/mL and at 0.26 ng/mL, respectively. The experiment was repeated three times and the mean percent inhibition was calculated (mean ± SD).

**Table 3:** In vitro anti-mycobacterial activity and combination index (CI) of compounds **1**, **2**, and **3** in a two-drug combination with cycloserine (CYC).

Compound	Concentration (µg/mL)	Anti-Mycobacterial Activity *Mtb* (H37Ra)			
		Compound Alone	Combination with 5.5 μg/mL CYC		
		% Inhibition	% Inhibition	CI ^a^	Outcome
**1**	50	53 ± 3.17	92 ± 4.22	0.9	Synergistic
	25	46 ± 2.88	85 ± 6.65	0.9	Synergistic
	12.5	31 ± 0.93	78 ± 4.14	0.8	Synergistic
	6.25	31 ± 0.93	78 ± 4.14	0.8	Synergistic
	3.12	6 ± 2.87	58 ± 4.16	0.7	Synergistic
	1.56	2 ± 0.51	51 ± 2.92	0.7	Synergistic
**2**	50	54 ± 5.06	96 ± 2.03	0.9	Synergistic
	25	17 ± 5.91	91 ± 2.25	0.6	Synergistic
	12.5	8 ± 2.01	79 ± 4.75	0.5	Synergistic
	6.25	5 ± 1.07	69 ± 8.46	0.6	Synergistic
	3.12	2 ± 1.21	54 ± 5.00	0.6	Synergistic
	1.56	0.5 ± 1.39	41 ± 3.39	0.8	Synergistic
**3**	25	49 ± 6.54	95 ± 2.58	0.9	Synergistic
	12.5	31 ± 5.83	91 ± 3.37	0.7	Synergistic
	6.25	21 ± 4.89	90 ± 4.84	0.6	Synergistic
	3.12	12 ± 6.12	88 ± 5.51	0.5	Synergistic
	1.56	7 ± 2.82	85 ± 5.13	0.5	Synergistic
	0.78	3 ± 2.18	72 ± 2.69	0.5	Synergistic
CYC	5.5	34 ± 4.63	-	-	-

^a^ CI < 1 (Synergistic), 1 (Additive). Anti-mycobacterial activity of compounds **1**, **2**, and **3** in combination with cycloserine (CYC) was determined at 50, 25, 12.5, 6.25, 3.12, and 1.56 µg/mL. The positive control drug CYC at 5.5 µg/mL was used. The experiment was repeated three times and the mean percent inhibition was calculated (mean ± SD).

**Table 4:** In vitro anti-mycobacterial activity and combination index (CI) of compounds **1**, **2**, and **3** in a three-drug combination with isoniazid and rifampicin.

Compound	Concentrations (µg/mL)		Anti-Mycobacterial Activity *Mtb* (H37Ra)		
		Compound Alone	Combination with 0.05 μg/mL INH and 0.00012 μg/mL RIF		
		% Inhibition	% Inhibition	CI ^a^	Outcome
**1**	25	52 ± 3.37	99 ± 1.39	1.0	Additive
	12.5	36 ± 3.02	98 ± 1.67	0.9	Synergistic
	6.25	15 ± 1.87	95 ± 0.56	0.7	Synergistic
	3.12	13 ± 3.20	94 ± 0.75	0.7	Synergistic
**2**	25	39 ± 2.16	97 ± 1.00	0.8	Synergistic
	12.5	18 ± 2.20	97 ± 0.78	0.7	Synergistic
	6.25	14 ± 1.56	93 ± 6.28	0.7	Synergistic
	3.12	13 ± 0.83	83 ± 3.38	0.6	Synergistic
**3**	25	49 ± 2.11	96 ± 1.06	1.0	Additive
	12.5	31 ± 2.13	95 ± 0.95	0.9	Synergistic
	6.25	28 ± 4.18	93 ± 2.17	0.8	Synergistic
	3.12	23 ± 3.38	89 ± 2.48	0.8	Synergistic
INH	0.05	38 ± 3.86	-	-	-
RIF	0.00012	15 ± 3.53	-	-	-

^a^ CI < 1 (Synergistic), 1 (Additive). CI of compounds **1**, **2**, and **3** in combination with isoniazid plus rifampicin was determined at concentrations of 25, 12.5, 6.25, and 3.12 µg/mL. The positive control drugs isoniazid at 0.05 µg/mL and rifampicin at 0.012 ng/mL were used. The experiment was repeated three times and the mean percent inhibition was provided (mean ± SD).

**Table 5:** In vitro anti-mycobacterial activity of compounds **1**, **2**, and **3** against *M. avium*.

Compound	Anti-Mycobacterial Activity ^a^ % Inhibition (Concentration, µg/mL) ^b^
	*M. avium*
**1**	50 ± 6.24 (200), 31 ± 2.53 (100), 19± 4.41 (50)
**2**	82 ± 2.52 (200), 36 ± 4.78 (100), 8 ± 5.89 (50)
**3**	98 ± 1.09 (200), 97 ± 1.59 (100), 86 ± 3.47 (50)
Clarithromycin ^c^	99 ± 2.11 (1)

^a^ Anti-mycobacterial activity was determined at concentrations of 200, 100, 50, 25, 12.5, 6.25, 3.12, and 1.56 µg/mL. ^b^ Average from three independent repeated experiments (mean ± SD). ^c^ Positive control TB drug.

**Table 6:** In vitro anti-mycobacterial activity and combination index (CI) of compound **3** in combination with clarithromycin against *M. avium*.

Compound	Concentration (µg/mL)	Anti-Mycobacterial Activity *M. avium*			
		Compound Alone	Combination with 0.09 µg/mL Clarithromycin		
		% Inhibition	% Inhibition	CI ^a^	Outcome
**3**	30	36 ± 2.03	95 ± 1.37	0.9	Synergistic
	20	20 ± 3.30	95 ± 1.87	0.7	Synergistic
	10	5 ± 2.14	65 ± 3.11	0.8	Synergistic
	5	3 ± 1.70	52 ± 2.57	0.9	Synergistic
Clarithromycin	0.09	48 ± 1.75	-	-	-

^a^ CI < 1 (Synergistic), 1 (Additive). CI of compound **3** in combination with clarithromycin was determined at 30, 20, 10, and 5 µg/mL concentrations. The positive control drug clarithromycin at 0.09 µg/mL was used. The experiment was repeated three times and the mean percent inhibition was calculated (mean ± SD).

## Data Availability

Data is available from the authors upon request.

## Sample Availability

Samples of compound **1** are available from the authors. Compounds **2** (Product number-H0562) and **3** (CAS number-219581-06-03) are available commercially from TCI America Inc., Portland, Oregon, USA, and Toronto Research Chemicals Inc., North York, ON, Canada, respectively.
